# Intravascular lithotripsy for coral reef aortic stenosis: a case series assessing feasibility, hemodynamic effects, imaging and clinical outcomes

**DOI:** 10.1186/s42155-026-00698-4

**Published:** 2026-06-17

**Authors:** Marios Platon Dimopoulos, Maria Papageorgiou, Georgios Nistikoulis, Panagiotis Kitrou, Dimitrios Karnabatidis, Konstantinos Katsanos

**Affiliations:** 1 Department of Interventional Radiology, University Hospital of Patras, Patras, 26504 Greece; 2 Department of Radiology, University Hospital of Patras, Patras, 26504 Greece

**Keywords:** Intravascular lithotripsy, Coral reef aorta, Fractional flow reserve, Endovascular treatment

## Abstract

Coral reef aorta (CRA) is defined by the presence of heavily calcified exophytic plaques that protrude into the aortic lumen. Open surgery remains the standard treatment but is associated with substantial morbidity, particularly in high-risk patients. Endovascular approaches, including intravascular lithotripsy (IVL), have emerged as less invasive alternatives. This case series aimed to evaluate the feasibility, safety, and hemodynamic impact of IVL combined with fractional flow reserve (FFR) in the treatment of calcified aortic stenoses. We present a case series of three patients with severe calcified abdominal aortic stenosis treated with IVL under FFR guidance. Pre- and post-procedural FFR measurements were used to assess lesion hemodynamic significance and treatment efficacy. All procedures were technically successful, with significant improvement in FFR values following IVL, indicating restoration of hemodynamic flow. One case was complicated by an aortic dissection, successfully managed with stent placement. In the remaining cases, IVL achieved adequate luminal gain without the need for stenting. At follow-up (up to 12 months), all patients demonstrated sustained clinical improvement and maintained vessel patency. IVL combined with FFR appears to be a feasible and effective minimally invasive approach for the management of heavily calcified aortic lesions. This strategy allows both morphological plaque modification and objective physiological assessment, potentially reducing the need for stent implantation. Larger studies are required to validate these findings.

## Introduction

Coral reef aorta (CRA) is a rare manifestation of advanced atherosclerosis, characterized by heavy calcifications causing severe aortic lumen narrowing and ischemic symptoms. Open surgery remains the standard treatment for CRA; however, a significant proportion of patients are not suitable candidates due to multiple comorbidities. In addition, surgery is associated with considerable morbidity, particularly in juxtarenal and paravisceral disease, including prolonged hospital stay, increased complication rates, and the need for repeat interventions.

Intravascular lithotripsy (IVL) offers a minimally invasive alternative, using low-pressure acoustic waves to fracture calcified plaques and restore vessel compliance while minimizing rupture risk [1–4]. By inducing microfractures within heavily calcified lesions, IVL improves vessel compliance and elasticity, thereby enhancing the effectiveness of subsequent balloon angioplasty. In heavily calcified, “coral reef”-type lesions, adequate stent expansion and optimal luminal gain are often not achievable. IVL facilitates modification of these calcified plaques, allowing proper vessel preparation and more controlled luminal expansion, potentially reducing the need for aggressive high-pressure dilation or extensive stent implantation strategies, while preserving the option for stenting at a later stage if needed. Combined with fractional flow reserve (FFR), IVL enables both morphological modification and physiological assessment of stenosis severity [5, 6].

We present three cases of calcified abdominal aortic stenoses treated with IVL under FFR guidance, highlighting its safety and effectiveness as a vessel-preserving option in complex aortic disease.

## Case presentation

Three patients with severe calcified abdominal aortic stenosis were treated with intravascular lithotripsy (IVL) under fractional flow reserve (FFR) guidance. FFR is a pressure-derived physiological index that quantifies the functional severity of arterial stenosis by comparing maximal blood flow with and without the lesion. It is defined as the ratio of distal pressure (Pd) to proximal pressure (Pa) during conditions of maximal hyperemia, when microvascular resistance is minimized. FFR is measured invasively using a pressure-sensor guidewire positioned across the lesion, with simultaneous proximal arterial pressure recording via the access sheath. FFR measurements were performed using a 0.014-inch pressure-sensor guidewire (COMET II Pressure Guidewire, Boston Scientific), advanced distal to the lesion, with intra-arterial papaverine (30 mg) administered distally to induce maximal hyperemia. Pharmacologic vasodilators (e.g., papaverine or adenosine) are administered to induce hyperemia and stabilize downstream resistance. The lowest Pd/Pa ratio during hyperemia represents the FFR value, with values of approximately ≤ 0.75–0.80 indicating hemodynamically significant disease by established coronary criteria [7]. FFR has been extensively validated in coronary arteries and has been shown to improve clinical outcomes and prolong survival by guiding revascularization decisions. In peripheral arteries, however, anatomical imaging alone often fails to reflect true lesion significance because of peripheral pulse amplification, especially in multilevel disease and in diabetes with medial wall sclerosis [8]. Peripheral circulation differs physiologically, with dominant systolic flow and higher, variable microvascular resistance affecting pressure measurements. Therefore, FFR may be particularly valuable in the aortoiliac and femoral arteries to provide lesion-specific functional assessment beyond angiography. Potentially, its use can help identify which lesions require intervention, optimize treatment strategies, and avoid unnecessary procedures in peripheral arterial disease [9–11].

### Case 1

**Fig. 1:**
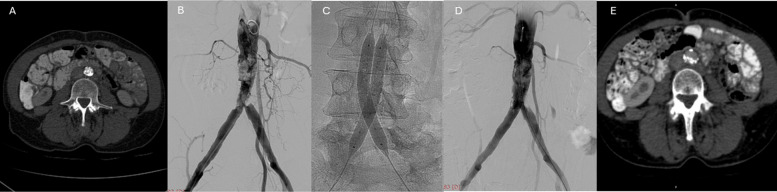
**A** Axial CTA demonstrating severe calcified aortic stenosis, **B** Baseline digital subtraction angiography (DSA) confirming concentric, heavily calcified narrowing of the aortic lumen, **C** Kissing intravascular lithotripsy (IVL) balloons positioned across the calcified aortic segment, **D** Final DSA showing restoration of the aortic lumen with preserved renal artery patency, **E** Axial follow-up CT demonstrating sustained luminal gain post-treatment

**Fig. 2:**
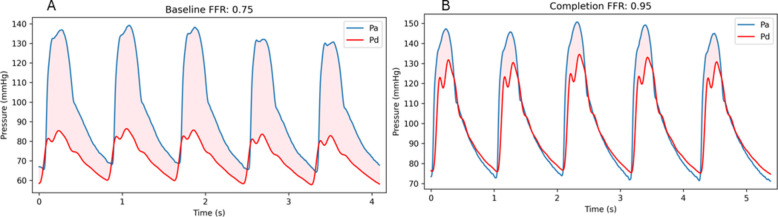
**A** Baseline FFR at hyperemia demonstrating a significant translesional pressure gradient, with distal vessel pressure (Pd) markedly lower than aortic pressure (Pa), resulting in a pathological FFR value of 0.75. **B** Post-intervention FFR at hyperemia demonstrating resolution of the translesional pressure gradient, with normalization of distal vessel pressure and an improved FFR value of 0.95

A 66-year-old woman with a history of active smoking presented with claudication (Rutherford-3). CT angiography (CTA) revealed up to 80% stenosis of the infrarenal abdominal aorta (Fig. 1A), confirmed by digital subtraction angiography (Fig. 1B). FFR values were 0.95 at rest and 0.75 at hyperemia, indicating hemodynamically significant stenosis (Fig. 2A).

The lesion was treated with IVL using two M5 + 8.0 × 60 mm Peripheral Intravascular Lithotripsy Catheters (135 cm), deployed using the kissing-balloon technique [4 atm, 50—60 Hz, 30 pulses, 2 cycles for a total of 60 pulses] (Fig. 1C). Post-procedural FFR improved to 0.95 at rest and hyperemia, confirming successful physiological treatment of the stenosis (Fig. 2B).

No peri-procedural complications occurred (Fig. 1D). At 3-month follow-up, the patient reported complete resolution of claudication, with unrestricted walking ability (Rutherford-0). Follow-up CTA at 5 months demonstrated a reduction in atherosclerotic plaque burden and a residual stenosis of 35% at the infrarenal abdominal aorta, sustained patency of the treated segment and preserved mesenteric artery flow (Fig. 1E). At 12-month clinical follow-up, the patient remained asymptomatic with no recurrence of claudication.

### Case 2

A 56-year-old man with a history of active smoking and treatment-resistant secondary hypertension presented with non-healing foot ulcers and rest pain, claudication (Rutherford-5). CTA demonstrated severe thoracoabdominal aortic stenotic lesions and left renal artery stenosis. Based on these findings, the patient underwent percutaneous angioplasty and stent placement in the left renal artery for hypertension management, with a balloon-expandable Dynamic Renal stent 7.0 × 19 mm (Biotronik AG), under FFR guidance. At 1-month follow-up CTA imaging revealed persistent severe abdominal aortic stenosis with an exophytic calcification extending to the visceral arteries origins, with similar lesions in both iliac and superficial femoral arteries.

The patient was scheduled for IVL for targeting the right iliac artery and the abdominal aorta. Pre-procedural FFR value was 0.69 at rest and 0.57 at hyperemia. Treatment was performed with a M5 + 7.0 mm × 60 mm Peripheral Intravascular Lithotripsy Catheter (135 cm) in the right common iliac artery, and two M5 + 7.0 mm Peripheral Intravascular Lithotripsy Catheters (135 cm) were deployed simultaneously in the abdominal aorta using kissing-balloon technique [4 atm, 50—60 Hz, 30 pulses, 2 cycles for a total of 60 pulses]. Post-procedural FFR was 0.94 at rest and 0.89 at hyperemia, confirming successful physiological treatment of the stenosis.

The procedure was completed successfully without complications. At 3-month follow-up, he reported marked improvement with only mild discomfort occurring after > 300 m walking and complete healing of foot ulcers (Rutherford-1). Follow-up Magnetic Resonance Angiography (MRA) demonstrated a patent left renal stent without residual stenosis, mild luminal irregularity of the suprarenal abdominal aorta, and preserved patency in the left common iliac artery and the peripheral segments of the renal and iliac arteries. At 12-month clinical follow-up, sustained clinical improvement was reported, with no recurrence of rest pain or ulceration.

### Case 3

**Fig. 3:**
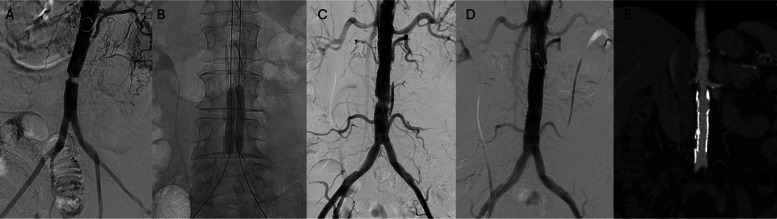
**A** Baseline DSA demonstrating extensive atherosclerotic disease with significant aortic stenosis, **B** Kissing intravascular lithotripsy (IVL) balloon technique applied across the lesion., **C** Post-IVL DSA revealing aortic dissection., **D** Covered stent implantation with restoration of luminal integrity and no residual stenosis., **E** Six-month follow-up CTA demonstrating no residual stenosis

A 61-year-old woman with a history of active smoking, hypertension, and dyslipidemia presented with bilateral claudication at 100 m, along with pain at rest, and toe cyanosis (Rutherford-4). CT angiography revealed both calcified and non-calcified atherosclerotic plaques in the abdominal aorta and iliac arteries, with up to 90% stenosis located approximately 3 cm above the aortic bifurcation. Diagnostic digital subtraction angiography confirmed the severity and extent of the lesion (Fig. 3A).

**Fig. 4:**
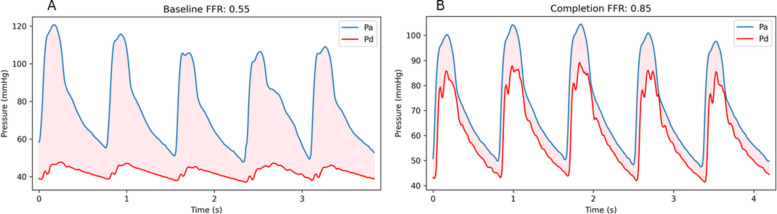
**A** Baseline FFR at hyperemia demonstrating a significant translesional pressure gradient, with distal vessel pressure (Pd) markedly lower than aortic pressure (Pa), resulting in a pathological FFR value of 0.55., **B** Post-intervention FFR at hyperemia demonstrating resolution of the translesional pressure gradient, with normalization of distal vessel pressure and an improved FFR value of 0.85

FFR values were 0.69 at rest and 0.55 at hyperemia (Fig. 4A). IVL was performed at the aortic bifurcation with two M5 + 8.0 × 60 mm Peripheral Intravascular Lithotripsy Catheters (135 cm) using the kissing-balloon technique (Fig. 3B). During balloon inflation, an aortic dissection developed (Fig. 3C), successfully managed with percutaneous angioplasty using two Abbott Vascular balloons (7.0 × 40 mm × 80 mm) followed by implantation of a 12 × 49 mm (GORE, VIABAHN Endoprosthesis) covered stent, restoring vessel integrity (Fig. 3D). Post-procedural FFR improved to 1 at rest and 0.85 at hyperemia (Fig. 4B).

At 3-month clinical follow-up, the patient reported complete resolution of symptoms (Rutherford-0). Follow-up CT angiography at 6 months demonstrated no residual stenosis of the treated aortic segment and stented area, with complete resolution of the previously identified stenosis (Fig. 3E). At 12-month clinical follow-up, the patient remained asymptomatic, with sustained clinical improvement and no evidence of symptom recurrence.

## Discussion

We describe three cases of severe calcified abdominal aortic stenoses at different anatomical levels: one involving the infrarenal abdominal aorta, one extending from the abdominal aorta toward the origins of the superior mesenteric and renal arteries, and one at the aortic bifurcation. In all cases, FFR confirmed hemodynamic lesion significance and IVL was used for vessel preparation.

In our case series, a single aortic dissection occurred, likely related to the heavily calcified lesion and the presence of underlying soft atherosclerotic plaque, which may have contributed to vessel wall fragility during balloon expansion, highlighting a potential complication that needs further investigation in future trials. Notably, in the other two cases IVL was effective without the need for stent implantation, suggesting that IVL may allow preservation of the native aorta and reduce the need for stenting in selected patients. Although imaging follow-up was limited to 5–6 months, extended clinical follow-up at 12 months confirmed sustained symptom improvement in all patients.

Open surgical reconstruction has long been the gold standard for treating heavily calcified aorto-iliac occlusive disease and coral reef aorta (CRA). However, surgery in the juxtarenal or paravisceral aorta is associated with considerable morbidity due to supraceliac clamping and extensive dissection. Currently, no atherectomy devices of sufficient size exist for the aorta, and thus no available tool is capable of achieving effective atherosclerotic debulking. IVL has recently emerged as a minimally invasive alternative, enabling vessel compliance modulation through sonic pressure waves that fracture calcified plaques at low inflation pressures (2–4 atm), thereby minimizing barotrauma and rupture risk.

Several reports have demonstrated the feasibility of IVL in large-caliber aortic segments. Donas et al. described an IVUS-guided, large-diameter (12 mm) low-pressure IVL using the Shockwave L6 balloon for a paravisceral CRA, achieving significant luminal gain, clinical improvement and six-month patency without stenting [1]. Similarly, Borioni et al. reported a stentless IVL procedure in a paravisceral CRA that restored distal pressures and triphasic femoral waveforms on follow-up, confirming effective plaque remodeling without the need for endograft implantation [2].

Troisi et al. presented a three-case series of juxtarenal and infrarenal CRA treated with kissing-balloon IVL balloons. Functional evaluation confirmed hemodynamic improvement post-intervention. Two of the three cases achieved durable luminal gain without stent placement, suggesting IVL can preserve the native aortic anatomy while reducing peri-procedural risks [3].

In the report by Elger et al., IVL achieved effective atherosclerotic debulking of the juxtarenal and visceral aorta, restoring vessel compliance and luminal gain. This facilitated optimal vessel preparation of both the renal artery and the adjacent aortic segment, enabling safe and precise stent deployment [4]. Another retrospective multicenter study by Elger et al., including 16 patients with paravisceral coral reef aorta presenting with claudication (94%), renal failure (56%), and mesenteric ischemia (25%), demonstrated 100% technical success, with no perioperative mortality or IVL-related complications [5].

In heavily calcified aortic lesions, angiographic evaluation after angioplasty may be insufficient due to calcium burden and projection limitations, necessitating supplementary assessment. FFR played a pivotal role as a diagnostic tool in the evaluation of stenosis, by providing hemodynamic data through computational fluid dynamics, offering a more comprehensive assessment of the functional significance of arterial stenoses [9]. Moreover, FFR assessment provided valuable insight into the technical outcome of the procedure by objectively confirming the degree of functional improvement achieved. Post-procedural FFR measurements objectively confirmed the substantial improvement in perfusion, thereby complementing angiographic outcomes with hemodynamic validation.

Collectively, these cases indicate that IVL can safely achieve meaningful luminal expansion in heavily calcified aortic lesions while avoiding the high barometric forces of traditional angioplasty. Integration of IVUS and FFR assessment may optimize procedural planning and confirm therapeutic efficacy.

In this case series, we evaluated the role of intravascular lithotripsy (IVL) and fractional flow reserve (FFR) in the management of calcified aortic stenoses. IVL appears to be a promising vessel-preserving strategy for selected patients with complex paravisceral or juxtarenal aortic disease, particularly when combined with physiological assessment tools such as FFR.

## Conclusion

In this case series, intravascular lithotripsy (IVL) demonstrated high technical success with an acceptable safety profile in the management of heavily calcified aortic stenoses. The adjunctive use of fractional flow reserve (FFR) provided objective hemodynamic assessment, confirming both lesion significance and post-treatment improvement. This combined approach may support more precise, individualized treatment strategies while potentially reducing the need for stent implantation in selected patients. Although current evidence remains limited, IVL appears to be a promising vessel-preserving option for complex aortic disease. Further studies are warranted to validate these findings and establish its long-term efficacy and safety.

## Data Availability

Data are available from the authors upon reasonable request.
